# Sarcopenia as a predictor of post-transplant tumor recurrence after living donor liver transplantation for hepatocellular carcinoma beyond the Milan criteria

**DOI:** 10.1038/s41598-018-25628-w

**Published:** 2018-05-08

**Authors:** Young Ri Kim, Sukhee Park, Sangbin Han, Joong Hyun Ahn, Seonwoo Kim, Dong Hyun Sinn, Woo Kyoung Jeong, Justin S. Ko, Mi Sook Gwak, Gaab Soo Kim

**Affiliations:** 1 Department of Anesthesiology and Pain Medicine, Samsung Medical Center, Sungkyunkwan University School of Medicine, Seoul, 06351 Korea; 2 Statistics and Data Center, Samsung Medical Center, Seoul, 06351 Korea; 3 Department of Medicine, Samsung Medical Center, Sungkyunkwan University School of Medicine, Seoul, 06351 Korea; 4 Department of Radiology, Samsung Medical Center, Sungkyunkwan University School of Medicine, Seoul, 06351 Korea

## Abstract

To evaluate the association between sarcopenia and tumor recurrence after living donor liver transplantation (LDLT) in patients with advanced hepatocellular carcinoma (HCC), we analyzed 92 males who underwent LDLT for treating HCC beyond the Milan criteria. Sarcopenia was defined when the height-normalized psoas muscle thickness was <15.5 mm/m at the L3 vertebra level on computed tomography based on an optimum stratification method using the Gray’s test statistic. Survival analysis was performed with death as a competing risk event. The primary outcome was post-transplant HCC recurrence. The median follow-up time was 36 months. There was a 9% increase in recurrence risk per unit decrease in height-normalized psoas muscle thickness. Twenty-six (36.1%) of 72 sarcopenic recipients developed HCC recurrence, whereas only one (5.0%) of 20 non-sarcopenic recipients developed HCC recurrence. Recurrence risk was greater in sarcopenic patients in univariable analysis (hazard ratio [HR] = 8.06 [1.06–16.70], p = 0.044) and in multivariable analysis (HR = 9.49 [1.18–76.32], p = 0.034). Greater alpha-fetoprotein and microvascular invasion were also identified as independent risk factors. Incorporation of sarcopenia improved the model fitness and prediction power of the estimation model. In conclusion, sarcopenia appears to be one of the important host factors modulating tumor recurrence risk after LDLT for advanced HCC.

## Introduction

Liver transplantation is an established therapeutic option to treat hepatocellular carcinoma (HCC) because it removes both the tumor and surrounding premalignant parenchymal tissues. However, severe graft shortage limits the candidacy for transplantation to patients with early stage HCC in whom the recurrence risk is relatively low and thus a lower probability of graft failure is predicted^1^. As one way to overcome the graft shortage, liver transplantation of grafts from living donors, so-called living donor liver transplantation (LDLT), allows more advanced HCC to be treated because living donors decide to donate at their own will and generally request permission to donate their grafts to a specific recipient without graft competition^2^. In this regard, better understanding of factors contributing to tumor recurrence of this highly invasive cancer is required to compensate for the high recurrence risk and improve post-transplant outcomes after LDLT.

In addition to tumor biology, patient functional status also affects tumor recurrence after treatment. Sarcopenia, or skeletal muscle deterioration, is frequently encountered in patients with end-stage liver disease, with a reported incidence ranging up to 70%, and is considered an important parameter indicating impaired functional status^2–5^. Previous studies have demonstrated that sarcopenia is associated with higher risk of HCC recurrence after liver resection^6–8^. Thus, we deduced that the risk of post-transplant recurrence of advanced HCC is affected by sarcopenia. In this study, we evaluated the relationship between sarcopenia and tumor recurrence after LDLT in patients with advanced HCC exceeding the Milan criteria (HCC beyond the Milan criteria).

## Results

### Characteristics of the subjects

The primary etiologies of HCC in the subjects were hepatitis B virus (n = 78), hepatitis C virus (n = 8), alcohol (n = 3), and unknown (n = 3). In the 92 patients, 91 presented with chronic liver cirrhosis, and 1 presented with acute-on-chronic liver failure. There were no recipients with acute liver failure or emergent surgery. Clinical characteristics of the two groups are described in Table 1. Body mass index was significantly lower in sarcopenic recipients than in non-sarcopenic recipients (23.8 vs. 25.5 kg/m^2^, p = 0.003). Accordingly, the proportion of recipients with <1.0% graft-to-recipient weight ratio was significantly lower in sarcopenic recipients (43.1% vs. 70.0%, p = 0.033). Age was significantly greater in sarcopenic recipients (54 vs. 51 years, p = 0.047). In terms of tumor characteristics, there were no significant differences between the two groups in alpha-fetoprotein level, tumor number/size, microvascular invasion, bile duct invasion, and non-tumor liver cirrhosis. Although statistical significance was not achieved, there was a trend toward a lower proportion of Edmonson grade III-IV (5.6% vs. 20.0%, p = 0.065) in sarcopenic recipients.

**Table 1:** Characteristics of patients included in the study.

	Sarcopenic (n = 72)	Non-sarcopenic (n = 20)	p
Graft factor			
Donor age (years)	28 (22–35)	31 (24–48)	0.082
Male donor	52 (72.2)	10 (50.0)	0.061
Graft-to-recipient weight ratio < 1.0%	31 (43.1)	14 (70.0)	0.033
Macrosteatosis ≥ 5%	35 (48.6)	7 (35.0)	0.280
Cold ischemia time (minutes)	85 (63–101)	85 (68–107)	0.450
Recipient factors			
Age (years)	54 (51–59)	51 (49–55)	0.047
Body mass index (kg/m^2^)	23.8 (21.8–25.5)	25.5 (24.6–28.2)	0.003
Diagnosed diabetes	25 (34.7)	8 (40.0)	0.663
MELD score ≥ 20	10 (13.9)	0 (0)	0.111
Sodium level (mmol/L)	139 (135–141)	140 (138–143)	0.027
Hepatic encephalopathy			
None	62 (86.1)	19 (95.0)	
Grade I–II (vs. none)	10 (13.9)	1 (5.0)	0.445
Grade III–IV	0	0	—
Refractory ascites	14 (19.4)	1 (5.0)	0.177
Pretransplant CRRT	0	0	—
Pretransplant tumor treatment history	56 (77.8)	14 (70.0)	0.555
High sensitive C-reactive protein (mg/L)	0.47 (0.17–1.16)	0.27 (0.10–0.69)	0.214
Neutrophil-to-lymphocyte ratio	2.01 (1.35–3.73)	1.52 (1.03–2.80)	0.085
Surgical factors			
Operative time > 10 hours	30 (41.7)	10 (50.0)	0.506
Perioperative RBC transfusion > 6 units	22 (30.6)	5 (25.0)	0.629
Tacrolimus trough concentration > 10 ng/mL	32 (44.4)	10 (50.0)	0.659
Tumor biology			
AFP (log-transformed ng/mL)*	3.98 (2.25–5.59)	2.87 (1.70–4.93)	0.196
Tumor number			0.663
Solitary	6 (8.3)	3 (15.0)	
2–3 (vs. solitary)	17 (23.6)	4 (20.0)	
>3 (vs. solitary)	49 (68.1)	13 (65.0)	
Tumor size (cm)	3.3 (2.5–5.3)	3.2 (2.3–5.0)	0.381
Microvascular invasion	42 (58.3)	14 (70.0)	0.344
Bile duct invasion	5 (4.7)	1 (5.0)	>0.99
Edmonson grade III-IV (vs. grade I-II)	4 (5.6)	4 (20.0)	0.065
Non-tumor liver cirrhosis	63 (87.5)	16 (80.0)	0.469

Data are presented as median (25th percentile, 75th percentile) or frequency (percent). AFP, alpha-fetoprotein; MELD, model for end-stage liver disease; RBC, red blood cell. CRRT, continuous renal replacement therapy. *P values were calculated for log-transformed values due to the skewed distribution.

### Survival analysis

**Figure 1:**
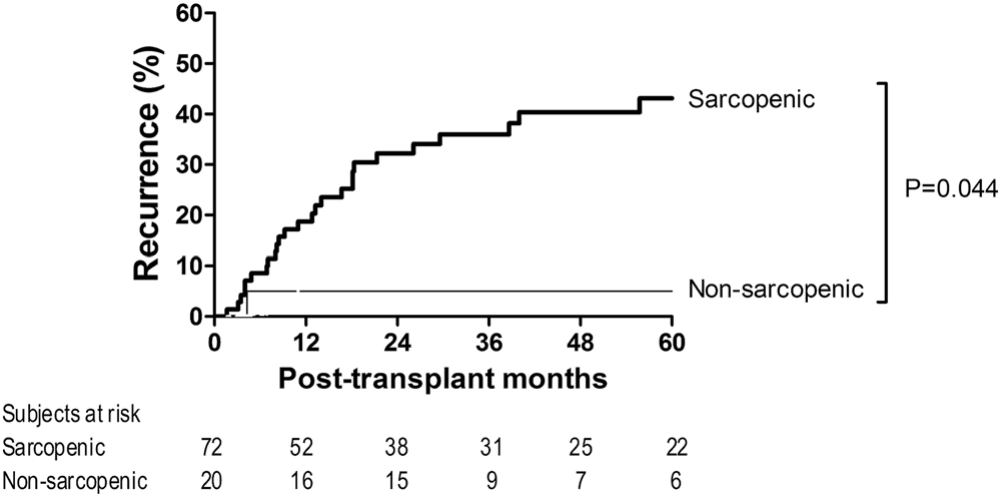
Cumulative recurrence probability according to the presence of sarcopenia. Curves are depicted until 5 years after transplantation because there was no recurrence after 5 years.

**Figure 2:**
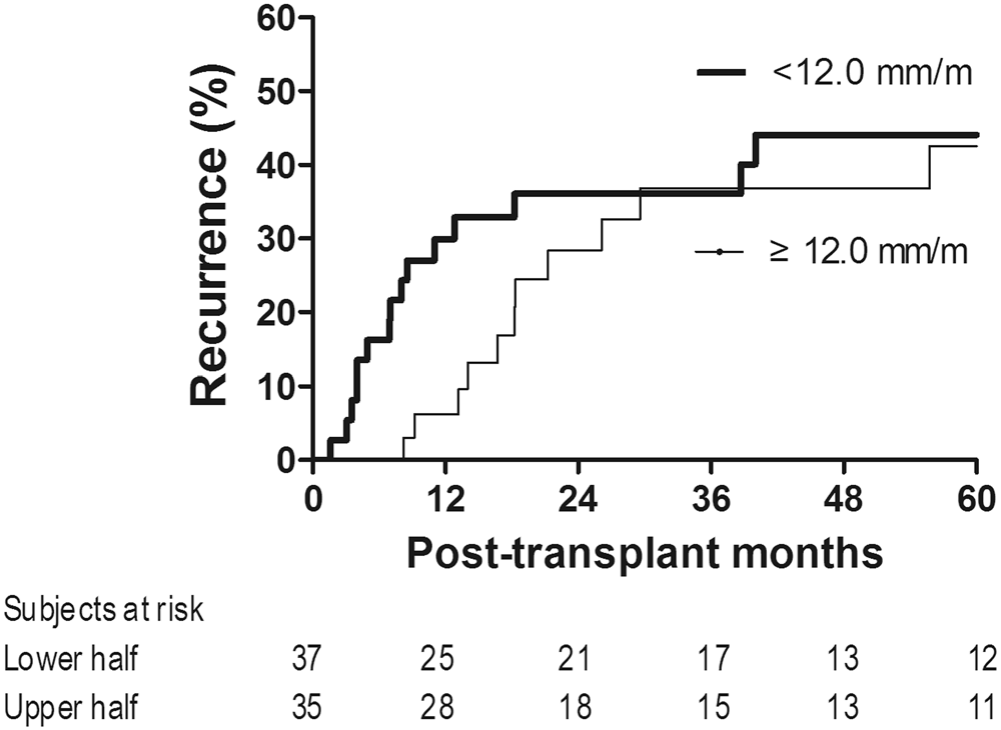
Cumulative recurrence probability of sarcopenic recipients stratified dichotomously into two groups.

**Figure 3:**
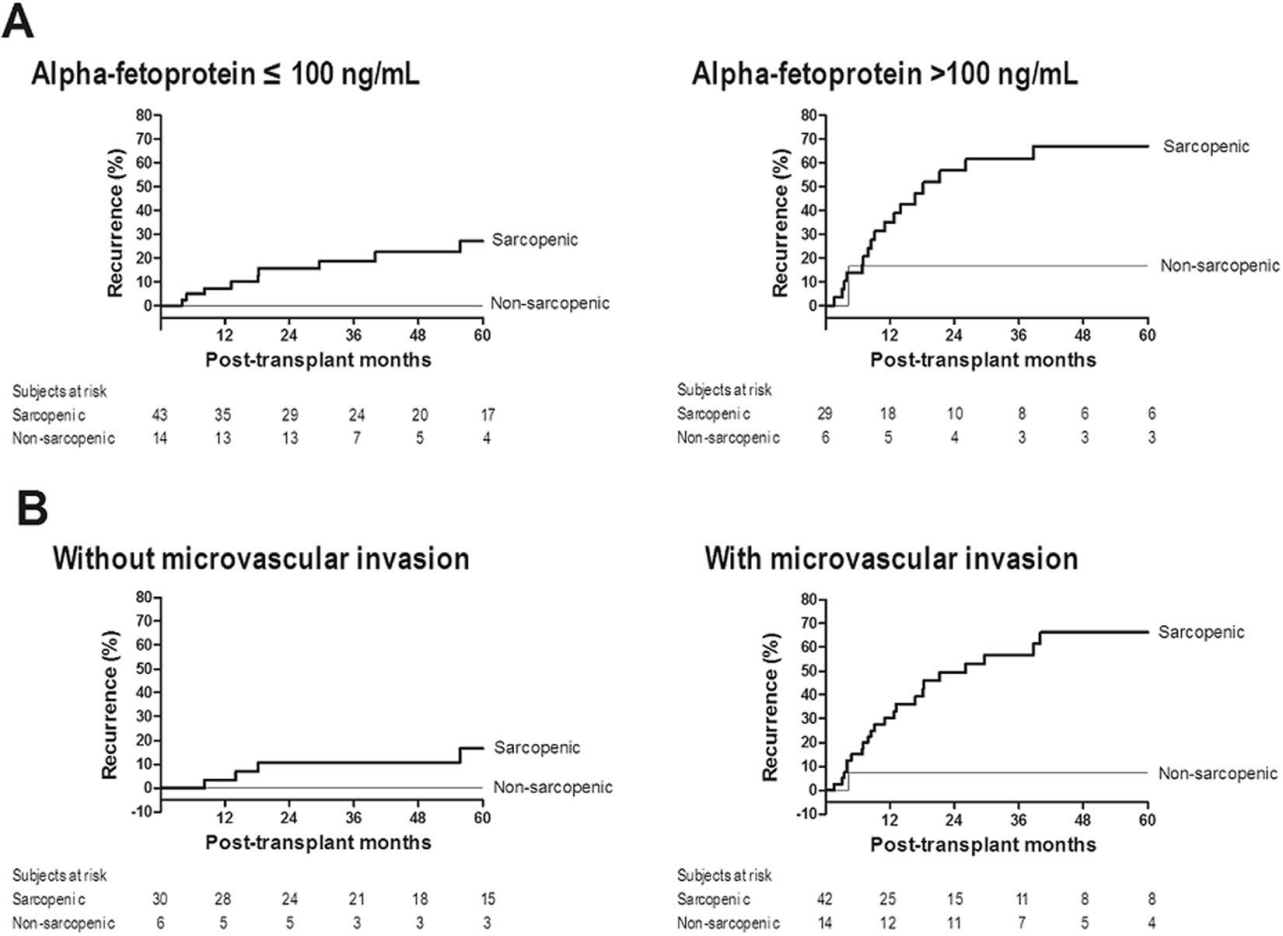
Cumulative recurrence probability according to the presence of sarcopenia in subgroups stratified by (**A**) alpha-fetoprotein level and (**B**) tumor microvascular invasion.

**Figure 4:**
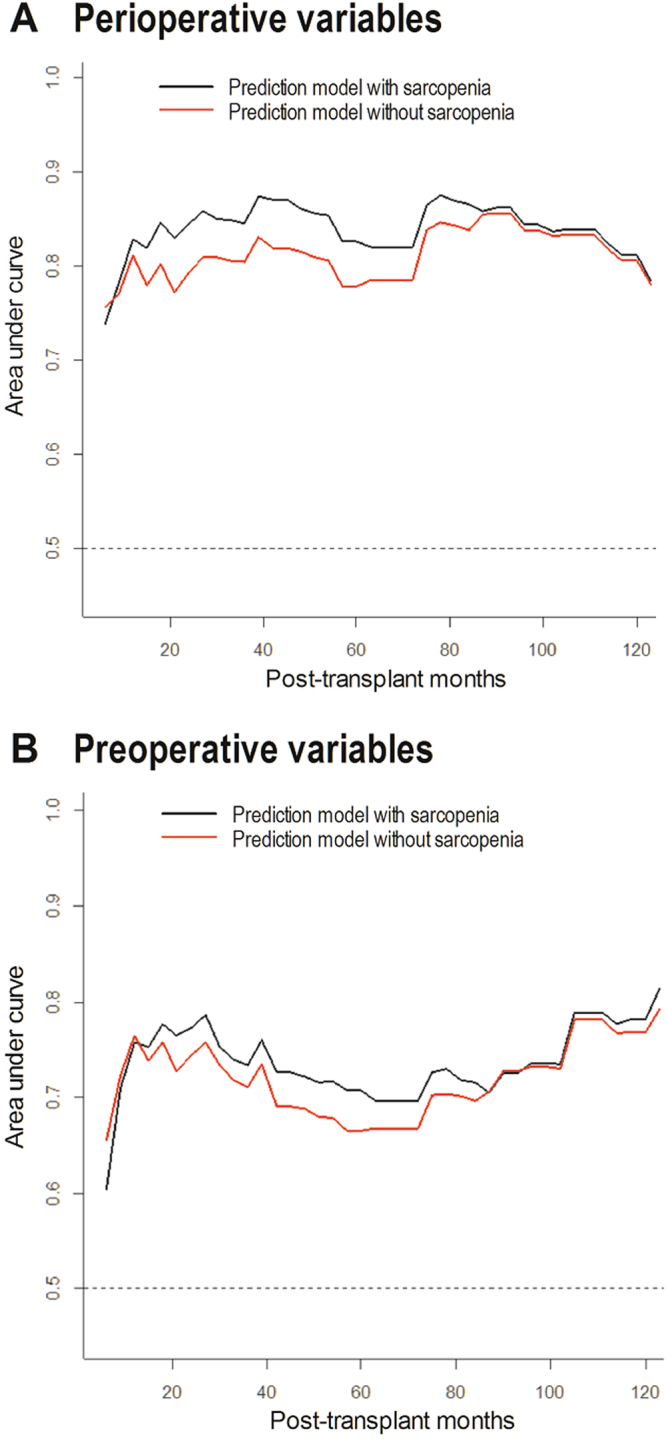
C-index of the prediction model for hepatocellular carcinoma recurrence. (**A**) sarcopenia plus alpha-fetoprotein and microvascular invasion vs. alpha-fetoprotein and microvascular invasion without sarcopenia and (**B**) sarcopenia plus alpha-fetoprotein vs. alpha-fetoprotein alone.

There were no recipients who were lost to follow-up without HCC recurrence. The median follow-up time was 36 (17–80) months. The median psoas muscle (PM) thickness was 13.1 (10.9–15.3) mm/m, and 72 (78%) patients showed sarcopenia. The continuous value of PM thickness was negatively associated with HCC recurrence risk with a marginal significance (coefficient = −0.089 [9% increase in recurrence risk per unit decrease in PM thickness], hazard ratio (HR) = 0.92 [0.83–1.01], p = 0.071). Twenty-seven (29.3%) recipients developed HCC recurrence: 26 of 72 (36.1%) sarcopenic recipients and one of 20 (5.0%) non-sarcopenic recipients (Fig. 1). The results of univariable analysis indicated that sarcopenia was significantly associated with HCC recurrence risk (HR = 8.06 [1.06–16.70], p* = *0.044). The non-significance of continuous PM thickness and significance of sarcopenia indicate that the clinical implications of PM thickness are not continuous, as shown in Supplementary Fig. S1. Graft-to-recipient weight ratio, recipient gender, body mass index, alpha-fetoprotein level, tumor size, and microvascular invasion, in addition to sarcopenia, were significantly associated with HCC recurrence (p < 0.05, Table 2). As shown in Table 3, multivariable analysis confirmed the significantly higher recurrence risk in patients with sarcopenia (HR = 9.49 [1.18–76.32], p = 0.034). Higher alpha-fetoprotein level (HR = 1.20 [1.03–1.39], p = 0.022) and microvascular invasion (HR = 5.30 [1.83–15.37], p* = *0.002) were also identified as independent risk factors. To further evaluate the relevance of the degree of sarcopenia, we graphically compared HCC recurrence risk of sarcopenic recipients with PM thickness of <12.0 mm/m and sarcopenic recipients with PM thickness of ≥12.0 mm/m, although statistical analysis could not be performed due to the lack of sufficient sample size. As shown in Fig. 2, there was a trend toward a high recurrence risk in sarcopenic recipients with lower PM thickness, particularly during the early period (2 years of transplantation), suggesting that the degree of sarcopenia is relevant in addition to the presence of sarcopenia. As shown in Fig. 3, there was a consistent trend toward higher recurrence risk in recipients with sarcopenia than in recipients without sarcopenia irrespective of the presence of other independent risk factors, indicating that there was no interaction effect of sarcopenia with alpha-fetoprotein or microvascular invasion on HCC recurrence and supporting the independence of sarcopenia. Moreover, the degree of model fitness was significantly better when the model includes sarcopenia in addition to alpha-fetoprotein and microvascular invasion than when the model only include alpha-fetoprotein and microvascular invasion (log-likelihood difference = 4.6, p = 0.032). As shown in Fig. 4A, time-dependent C-index was >0.80 in most follow-up period, indicating a strong discrimination ability, and it was graphically greater when the model includes sarcopenia although statistical difference cannot be calculated due to the insufficient sample size. When considering only two preoperative variables (sarcopenia and alpha-fetoprotein), incorporation of sarcopenia with alpha-fetoprotein significantly increased the model fitness (log-likelihood difference = 4.0, p = 0.046). Time-dependent C-index was graphically greater when the model incorporated sarcopenia with alpha-fetoprotein (Fig. 4B).

**Table 2:** Descriptive statistics and univariable analysis.

	HR (95% CI)	p
Graft factor		
Donor age (years)	0.99 (0.95–1.03)	0.550
Male donor	1.78 (0.70–4.56)	0.230
Graft-to-recipient weight ratio < 1.0%	0.48 (0.22–1.07)	0.071
Macrosteatosis ≥ 5%	0.89 (0.42–1.90)	0.770
Cold ischemia time (minutes)	0.99 (0.98–1.01)	0.320
Recipient factors		
Age (years)	1.02 (0.95–1.09)	0.680
Male	—	—
Body mass index (kg/m^2^)	0.84 (0.74–0.96)	0.010
Diagnosed diabetes	1.09 (0.51–2.34)	0.820
MELD score ≥ 20	0.87 (0.26–2.87)	0.820
Sodium level (mmol/L)	1.06 (0.98–1.14)	0.160
Hepatic encephalopathy		
None		
Grade I-II (vs. none)	0.83 (0.25–2.75)	0.757
Grade III–IV	0	—
Refractory ascites	0.52 (0.16–1.65)	0.260
Pretransplant CRRT	—	—
Pretransplant tumor treatment history	3.92 (0.90–17.01)	0.068
High sensitive C-reactive protein (mg/L)	1.06 (0.92–1.22)	0.419
Neutrophil-to-lymphocyte ratio	1.03 (0.90–1.17)	0.680
Sarcopenia	8.06 (1.06–16.7)	0.044
Surgical factors		
Operative time >10 hours	0.57 (0.25–1.31)	0.190
Perioperative RBC transfusion > 6 units	0.52 (0.20–1.35)	0.180
Tacrolimus trough concentration > 10 ng/mL	2.14 (0.99–4.62)	0.054
Tumor biology		
AFP (log-transformed ng/mL)*	1.30 (1.12–1.52)	0.001
Tumor number		
Solitary		
2–3 (vs. solitary)	0.89 (0.16–5.05)	0.890
>3 (vs. solitary)	1.55 (0.34–7.09)	0.570
Tumor size*	1.25 (1.14–1.37)	<0.001
Microvascular invasion	5.05 (1.80–14.19)	0.002
Bile duct invasion	2.34 (0.70–7.86)	0.168
Edmonson grade III–IV (vs. grade I–II)	2.03 (0.56–7.29)	0.280
Non-tumor liver cirrhosis	0.50 (0.19–1.34)	0.170

Data are presented as median (25th percentile, 75th percentile) or frequency (percent). Sarcopenia was defined based on transverse psoas muscle thickness. AFP, alpha-fetoprotein; MELD, model for end-stage liver disease; RBC, red blood cell. CRRT, continuous renal replacement therapy. *Hazard ratios and P values were calculated for log-transformed values due to skewed distribution.

**Table 3:** Multivariable analysis.

	Coefficient	HR (95% CI)	p
Sarcopenia	2.25	9.49 (1.18–76.32)	0.034
Alpha-fetoprotein (log-transformed ng/mL)	0.18	1.20 (1.03–1.39)	0.022
Microvascular invasion	1.67	5.30 (1.83–15.37)	0.002

Variables with p < 0.05 during univariable analysis were included in the model and backward stepwise selection was used for generating the final model. Sarcopenia was defined based on transverse psoas muscle thickness.

**Figure 5:**
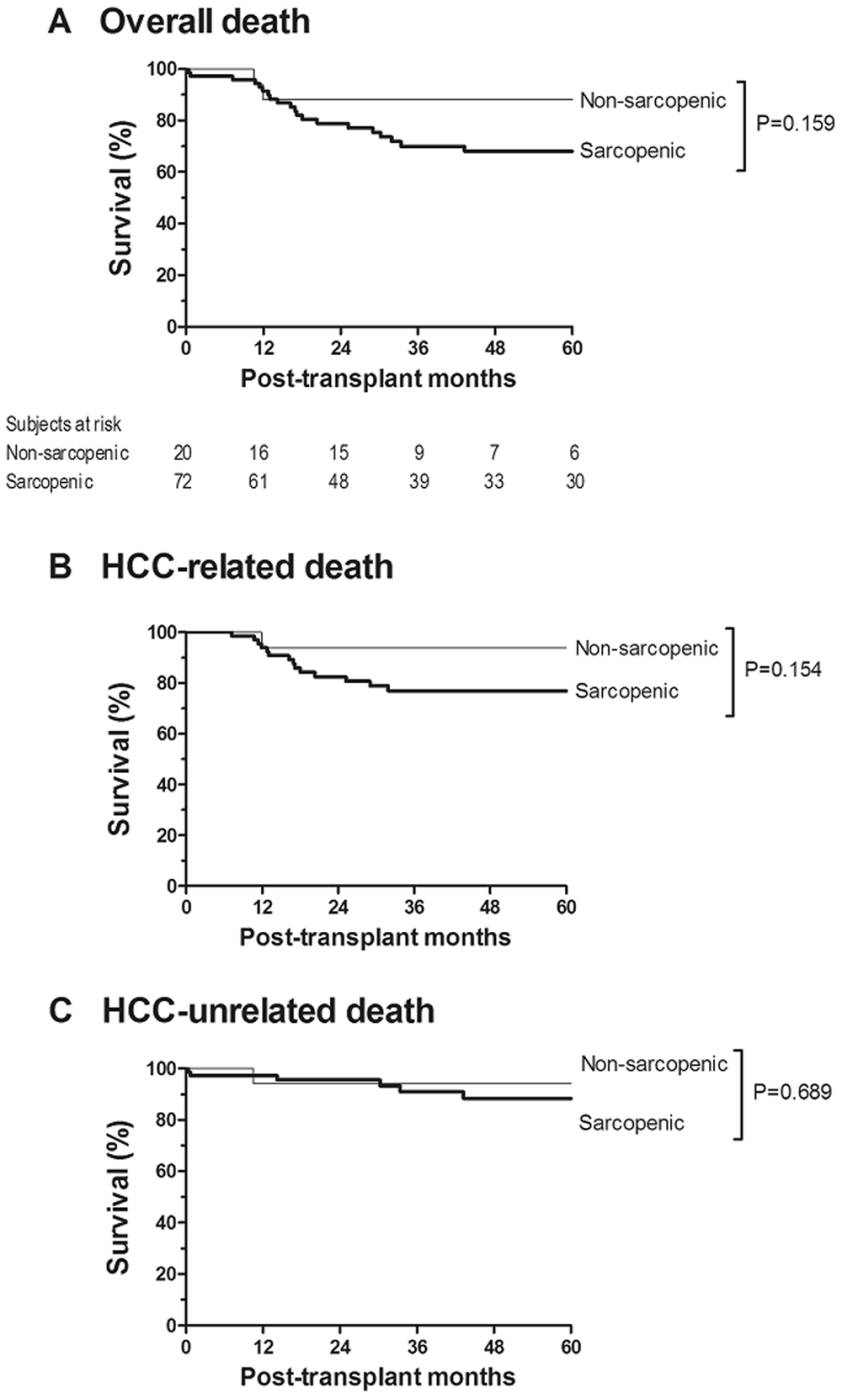
Survival probability according to the presence of sarcopenia. (**A**) Overall death, (**B**) HCC-related death, and (**C**) HCC-unrelated death.

Among the 23 recipients who died, 16 died of HCC recurrence and 7 died from HCC-unrelated causes. As shown in Fig. 5, there was a trend toward higher HCC-related death risk in sarcopenic patients (HR = 3.89 [0.51–29.49, p = 0.154), whereas the risk of HCC-unrelated death was comparable irrespective of the presence of sarcopenia (HR = 1.53 [0.19–12.76], p = 0.689). This finding indicates that sarcopenia-related HCC recurrence is an important cause of death in patients undergoing liver transplantation for advanced HCC.

## Discussion

This study demonstrated that sarcopenia was independently associated with tumor recurrence after LDLT in male patients with advanced HCC. The predominant cause of death in sarcopenic recipients was post-transplant HCC recurrence. In addition, the current study suggested a possibility that there is a significant degree of heterogeneity in tumor recurrence risk even in advanced HCC exceeding the Milan criteria, which is in agreement with recent studies suggesting the feasibility of using wider selection criteria for liver transplantation for advanced HCC^2,9,10^. That is, post-transplant HCC recurrence risk can be predicted more precisely based on skeletal muscle mass and alpha-fetoprotein, which may help avoid unnecessary discarding of LDLT due to the concern of high recurrence risk or help the more cautious selection of recipients who would stand to benefit from LDLT considering the potential donor risk^1^. Also, the better prediction may provide potential advantages to help physicians guide their perioperative clinical decision to adjust recurrence risk in terms of living donor selection, autologous transfusion^11^, immunosuppression^12^, and the use of an adjuvant treatment such as sorafenib^13^.

The mechanism underlying the association between sarcopenia and HCC recurrence is unclear, but may be explained by the tumor microenvironment (inflammation and immunity) and cytokine (myokines and adipokines)^14^. HCC recurrence arises from cancer cells present in the circulation or in micrometastatic colonies at the moment of transplantation and those cells can complete the metastatic cascade and reinitiate growth to form metastases within a few weeks^15^. Moreover, the intraoperative and early post-transplant period is a time window vulnerable to metastasis because patients experience surgery-induced stress, inflammation, platelet activation, transfusion, and immunosuppression. Thus, the interaction between HCC cells and the microenvironment during this early period is important. As the largest endocrine organ in the body, skeletal muscle produces and releases various physiologically important cytokines and proteins (named myokines) that contribute to the tumor microenvironment^16^. Myokines counteract the harmful effects of proinflammatory adipokines; accordingly, muscle waste tilts the balance to the proinflammatory pathways and results in systemic inflammation as well as immune disturbance. For instance, muscle waste results in the increase in IL-6, an inflammatory cytokine which is thought to increase the risk of HCC developement^14,17^. On the other hand, muscle waste results in the decrease in the production of IL-15, which plays an important role in inhibiting adipose tissue and reversing insulin resistance, and the proliferation and development of natural killer (NK) cells^16,18^. Moreover, the increase in adipokines, such as leptin, tumor necrosis factor (TNF-a), and IL-6, reduces the survival of NK cells^14,19^. These interrelations between myokines and adipokines may be related to the recurrence of HCC in sarcopenic patients because inflammation and escape from the host immune system are both important components for metastasis^16^. In short, it can be deduced that preoperative sarcopenia that persists during the vulnerable early post-transplant period induces a more favorable microenvironment for metastasis of advanced HCC cells via increasing inflammation and decreasing immunity and promotes tumor recurrence. Clinical evidence for the importance of body composition (sarcopenia, sarcopenic obesity, visceral obesity, or bone loss) on HCC recurrence after liver resection^6–8^ or liver transplantation^20,21^ supports the findings of the current study.

Multiple factors produce sarcopenia in patients with chronic liver disease, including loss of appetite, nutrient malabsorption, hypermetabolism, poor hepatic glyconeogenic capacity, and complications such as chronic fatigue, portal hypertension, ascites, and encephalopathy^3,22^. The roles of potential mediators of the liver-muscle axis in cirrhosis, such as ammonia, testosterone, and growth hormone, are under investigation^22^. Cancer cachexia also contributes to sarcopenia^23^, which in part explains the high incidence of sarcopenia in the current cohort of patients with advanced HCC. Based on the available evidence of contributors to sarcopenia, various strategies to increase skeletal muscle mass and improve outcomes in the non-transplant and post-transplant populations have been evaluated. These strategies include supplemental nutrition, physical activity, treating refractory ascites, ammonia reduction, and molecular targeted strategies such as myostatin antagonists and mitochondrial protective agents^22^.

Despite the high prevalence of sarcopenia and its association with clinical outcomes in cirrhotic patients, it is not efficiently incorporated into the clinical practice. One main reason is the lack of readily available, easy-to-measure, and objective parameters representing skeletal muscle mass^5^. Conventional methods for skeletal muscle measurement include anthropometric measurements, laboratory parameters (e.g. albumin), bioelectrical impedance, dual-energy X-ray absorptiometry, and magnetic resonance spectroscopy. Unfortunately, the applicability of these methods is limited in cirrhotic patients due to the characteristics of cirrhosis such as edema, hypoalbuminemia, and ascites^3^. However, recent studies have shown that computed tomography (CT) objectively measures skeletal muscle mass and predicts the prognosis in cirrhotic patients^4,5,24^. In particular, a recent study demonstrated that CT can be used as a reliable tool to measure PM thickness and diagnose sarcopenia even in ascitic patients and the authors explained that its reliability is attributed to the anatomical distinction between ascites and the PM, which is surrounded by retroperitoneal fat tissue and vertebra and is not affected by ascites^25^. Of note, PM thickness, which has been validated in the current and recent studies^24,25^, can be easily measured in a picture archiving and communication system without specific software, allowing it to be more applicable as a daily clinical practice.

The recurrence probability at 5 years after transplantation was 35.9% (21.6%–50.4%) in our cohort. As demonstrated in the current and previous studies, the probability of HCC recurrence after liver transplantation for HCC beyond the Milan criteria may vary by cohort according to the respective proportion of high risk and low risk recipients. A previous study stratified a validation cohort of 100 patients with HCC beyond the Milan criteria into high (n = 35) and low (n = 65) risk of HCC recurrence according to the alpha-fetoprotein model^10^. The recurrence probability at 5 years post-transplantation was 7.7%–14.4% in the low risk group and 46.3%–47.6% in the high risk group, respectively. Our cohort consisted of 44 low risk recipients and 48 high risk recipients according to the alpha-fetoprotein model and our recurrence probability is in agreement with the calculated probability based on the alpha-fetoprotein model.

Our study model has several advantages in terms of data quality. First, this study included a homogeneous LDLT population. All recipients received right hemi-liver graft without variations through a single transplant team. All recipients underwent an elective surgery without emergent conditions or acute deterioration. Only 4 patients had a MELD score >30. Accordingly, thorough perioperative anesthetic and surgical care could be performed in an unhurried fashion strictly based on the standardized institutional protocols. Second, recurrence evaluation was rigorously performed and there were no follow-up losses; thus, the data regarding the time-to-recurrence and time-to-death were highly reliable. Third, biasing effects from HCC-unrelated death was another concern because HCC-unrelated death excludes patients from the risk set and changes the recurrence probability. Moreover, our previous research demonstrated that the impact of a clinical variable on HCC-related death versus HCC-unrelated death can be different^26,27^. Thus, the Fine and Gray model was used by accounting for the competing risk of HCC-unrelated death instead of the standard Cox model. This issue was relevant because sarcopenia could also negatively influence the post-transplant outcomes of non-HCC patients and could confound the effects on HCC recurrence^28–30^. Fourth, even though the number of recurrences is apparently small, the power of this study to compare the hazard ratios between the sarcopenic group and non-sarcopenic group is >90% under the significance level of 5%. The power was calculated after adjustment with variance inflation factor (1/0.95) between sarcopenia and two variables (alpha-fetoprotein and microvascular invasion) for the multivariable model expecting the HR 9.49^31^.

This study has several limitations. First, due to its retrospective nature, a direct cause and effect relationship between sarcopenia and HCC recurrence remains unknown. In particular, sarcopenia is a component part of cancer cachexia^23^; thus, it is unclear whether sarcopenia directly impacts the metastasis of HCC cells or if sarcopenia is an epiphenomenon. In this regard, the effects of anti-sarcopenia treatment on HCC recurrence are also unclear and warrant future research. Second, as the first to evaluate the relationship between sarcopenia and post-transplant recurrence of HCC beyond the Milan criteria, this study set the cutoff PM thickness at 15.5 mm/m based on optimal stratification within our data because there are no reference values. Additional research is warranted to validate the PM thickness applicable in clinical practice. In particular, the cutoff values might differ by ethnicity: a previous study of patients listed for transplantation in France set the cutoff at 16.8 mm/m^24^; in contrast, another study of ascitic patients in South Korea set the cutoff at 14.0 mm/m^25^. Sex also affects the amount of skeletal muscle mass^29^; thus, the cutoff value in the current study cannot be translated to female recipients. Third, some tumor cells remain dormant for a long period at the secondary site and initiate growth later^15^. In this setting, metastasis occurs over an extended period of time. Thus, post-transplant long-term change in PM thickness may influence HCC recurrence^32^.

In summary, the risk of tumor recurrence after LDLT for treating HCC beyond the Milan criteria was significantly higher in patients with psoas muscle wasting. Despite the methodological limitation, our findings suggest that sarcopenia is an important prognostic factor for post-transplant tumor recurrence of advanced HCC.

## Materials and Methods

### Subjects

We screened the records of 140 male recipients who underwent a first adult-to-adult LDLT between May 2002 and May 2014 in our hospital (200th and 1500th LT cases) and were pathologically diagnosed as HCC beyond the Milan criteria without macrovascular invasion. None of the donor livers were procured from prisoners. We only included male recipients because reference values for PM thickness can differ by sex^4^. We excluded 3 recipients who underwent cyclosporine-based immunosuppression due to possible tacrolimus toxicity and 2 recipients due to missing data. Among the remaining 135 recipients, only those with utilizable cross-section PM images at the third lumbar vertebra (L3) with CT within 2 months before surgery were included because a 2-month duration is considered enough for muscle composition to be changed^4,7,33^. Accordingly, we excluded 15 recipients in whom the lowest CT image did not reach the L3 level, 20 recipients who had only outside CT images, and 8 recipients who underwent CT earlier than 2 months before surgery. The results of the comparison between the 92 recipients with an available CT image and 43 recipients without an available CT image are described in Supplementary Table S2, implying a low risk of selection bias from the exclusion of 43 recipients.

### Data collection

Data were obtained from computerized medical records and a liver transplantation database (prospectively collected). Recipients were followed until the end of the study in March 2015. Histology results of the removed liver were obtained from the postoperative pathology report. No patients received radiation or adjuvant chemotherapy for tumor prophylaxis after transplantation. The recurrence of HCC was defined on the basis of radiological evidence. Recipients were monitored regularly by measurement of serum alpha-fetoprotein level every 1–2 months, and chest radiography and abdominal ultrasonography or CT was used every 3 to 6 months to check for evidence of recurrence. When tumor recurrence was suspected, CT, magnetic resonance imaging, bone scan, or positron emission tomography scan was used to detect intrahepatic/extrahepatic metastasis. The Institutional Review Board of Samsung Medical Center approved this study (SMC 2015-05-002-001) and waived the requirement for written informed consent. All procedures applied to this study were performed in accordance with the relevant guidelines and regulations.

### Muscle mass quantification

**Figure 6:**
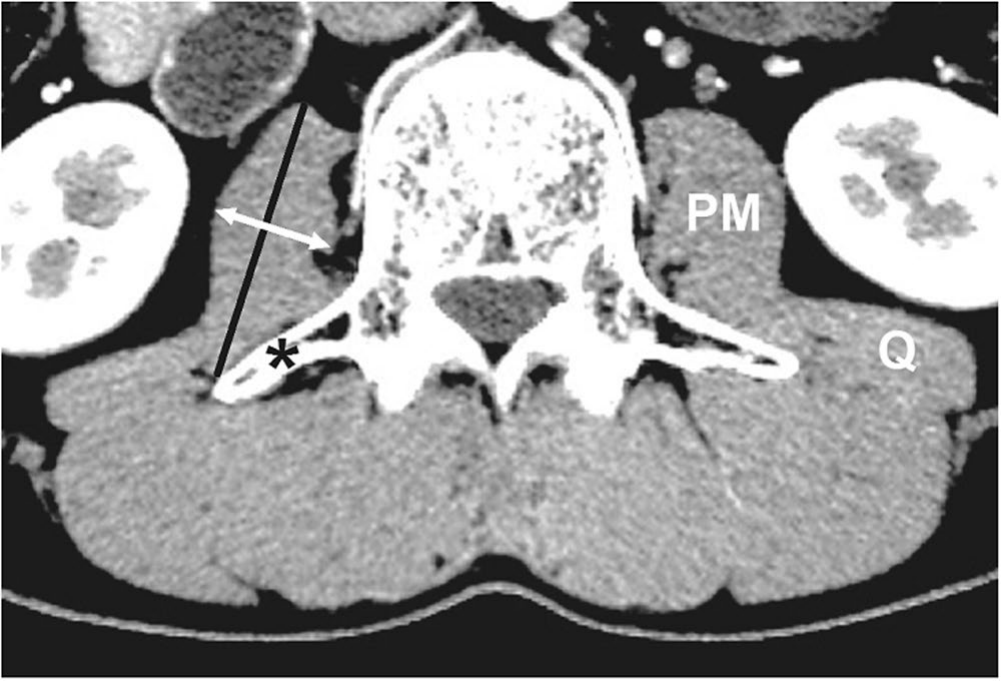
Psoas muscle measurement with a cross-sectional CT image. White arrow, psoas muscle thickness (PMT); black line, antero-posterior axis of the psoas muscle; asterisk, the transverse process of the 3rd lumber vertebra; Q, quadratus lumborum muscle.

PM thickness, the largest diameter perpendicular to the longest antero-posterior axis of the PM including both the psoas major and psoas minor (Fig. 6), was used as a surrogate marker of PM mass based on recent studies demonstrating the importance of PM thickness on clinical outcomes in cirrhotic patients^24,25^. PM thickness was measured on the right PM of a single axial CT image at the level of the L3 transverse process based on previous studies demonstrating that PM mass measured by CT at the L3 level corresponds to whole abdominal skeletal muscle mass and whole body skeletal muscle mass^4,33^. PM thickness was normalized to stature by dividing with height (mm/m)^4,24^. A single investigator (Y.L.K.) uniformly evaluated all CT images on a picture archiving and communication system (PathSpeed, GE Healthcare, Chicago).

### Liver transplant indication

Acceptance criteria for liver donation were age ≤65 years, body mass index <35 kg/m, macrosteatosis ≤30%, and residual liver volume ≥30%. LT for HCC diagnosed to exceed the Milan criteria during preoperative evaluations was performed upon the patient’s and donor’s request after thorough explanation of the higher recurrence probability^26^. Extrahepatic metastasis or macrovascular invasion detected in preoperative imaging evaluations was a contraindication for transplantation.

### Surgical and anesthetic managements

Surgical and anesthetic management for donors^34^ and recipients^35^ was performed based on standardized institutional protocols, as described previously. In short, all grafts consisted of segment 5–8 excluding the middle hepatic vein trunk. Graft implantation was performed using the piggyback technique. Blood transfusion was strictly controlled by means of the restrictive and prophylactic transfusion strategy^35^. A blood salvage procedure was routinely used despite the presence of HCC for reducing the requirement for allogeneic red blood cell transfusion and its fatal complications^11^. Immunosuppression was performed using the quadruple regimen as described previously^26^. Induction consisted of bolus steroid injection, which was tapered by the third month, and basilliximab. Tacrolimus and mycophenolate mofetil were used as maintenance therapy. Tacrolimus treatment was initiated on postoperative day 3, and the blood level was adjusted to maintain a daily measured trough plasma concentration of 10 ng/mL during the first month, which was reduced to 5–8 ng/mL thereafter.

### Variables and statistical analysis

The primary outcome was overall (intrahepatic or extrahepatic) HCC recurrence after transplantation. Post-transplant death was considered the competing risk event and survival analysis was performed using the Fine and Gray model^36^. The cutoff PM thickness for categorizing patients as high and low risk of HCC recurrence was set at 15.5 mm/m based on optimal stratification by means of the Gray’s test statistic (Supplementary Fig. S1)^27^. The cutoff values for mean trough tacrolimus concentration during the first month after transplantation were set at 10 ng/mL^12^. Variables with p < 0.05 during univariable analysis were included in multivariable analysis and backward stepwise selection was used for generating the final multivariable model^37^. Multicollinearity was tested by means of the variance inflation factor. Interaction effect between sarcopenia and other contributors for HCC recurrence were graphically tested by means of subgroup analysis while the cutoff value for alpha-fetoprotein was set at 100 ng/mL^10^. The benefit of incorporating sarcopenia into the prediction model was statistically tested using the likelihood test and graphically tested using the time-dependent receiver operating characteristic curve. Continuous variables are summarized as median (25th percentile, 75th percentile), and categorical variables are presented as frequency (%). All reported *P* values were two-sided and p < 0.05 was considered statistically significant. Analyses were performed using SPSS 20.0 (IBM Corp., Chicago, IL), R 3.0.2 (R Development Core Team, Vienna, Austria; http://www.R-project.org/), or SAS 9.4 (SAS Institute, Cary, NC).

### Data availability

The datasets generated and/or analyzed during the current study are available from the corresponding author on reasonable request.

## Electronic supplementary material

Supplementary materials
