# Supplementary material for: Sarcopenia as a predictor of post-transplant tumor recurrence after living donor liver transplantation for hepatocellular carcinoma beyond the Milan criteria
Source: Sci Rep. 2018 May 8;8:7157. doi: 10.1038/s41598-018-25628-w (PMC5940915; doi:10.1038/s41598-018-25628-w)
Supplement: Supplementary file 1 — Supplementary materials [file 41598_2018_25628_MOESM1_ESM.doc]

**Sarcopenia as a predictor of post-transplant tumor recurrence after living donor liver transplantation for hepatocellular carcinoma beyond the Milan criteria**

**Young Ri Kim^1^, Sukhee Park^1^, Sangbin Han^1^,*, Joong Hyun Ahn^2^, Seonwoo Kim^2^,**

**Dong Hyun Sinn^3^, Woo Kyoung Jeong^4^, Justin S. Ko^1^, Mi Sook Gwak^1^, Gaab Soo Kim^1^**

^1^Department of Anesthesiology and Pain Medicine, ^3^Medicine, and ^4^Radiology, Samsung Medical Center, Sungkyunkwan University School of Medicine, Seoul 06351, Korea

**^2^**Statistics and Data Center, Samsung Medical Center, Seoul 06351, Korea

Correspondence: Sangbin Han, MD, PhD, Department of Anesthesiology and Pain Medicine, Samsung Medical Center, Sungkyunkwan University School of Medicine, 81 Irwon-ro, Gangnam-gu, Seoul 06351, Korea. Tel.: +82-10-3410-2470; Fax: +82-2-3410-0361; E-mail: [hans5@skku.edu](mailto:sangbin.han@samsung.com)

**Table of Contents for Supplementary Materials**

| **Supplementary Figure S1** | **2** |
| --- | --- |
| **Supplementary Table S1** | **3** |

**Supplementary Materials**


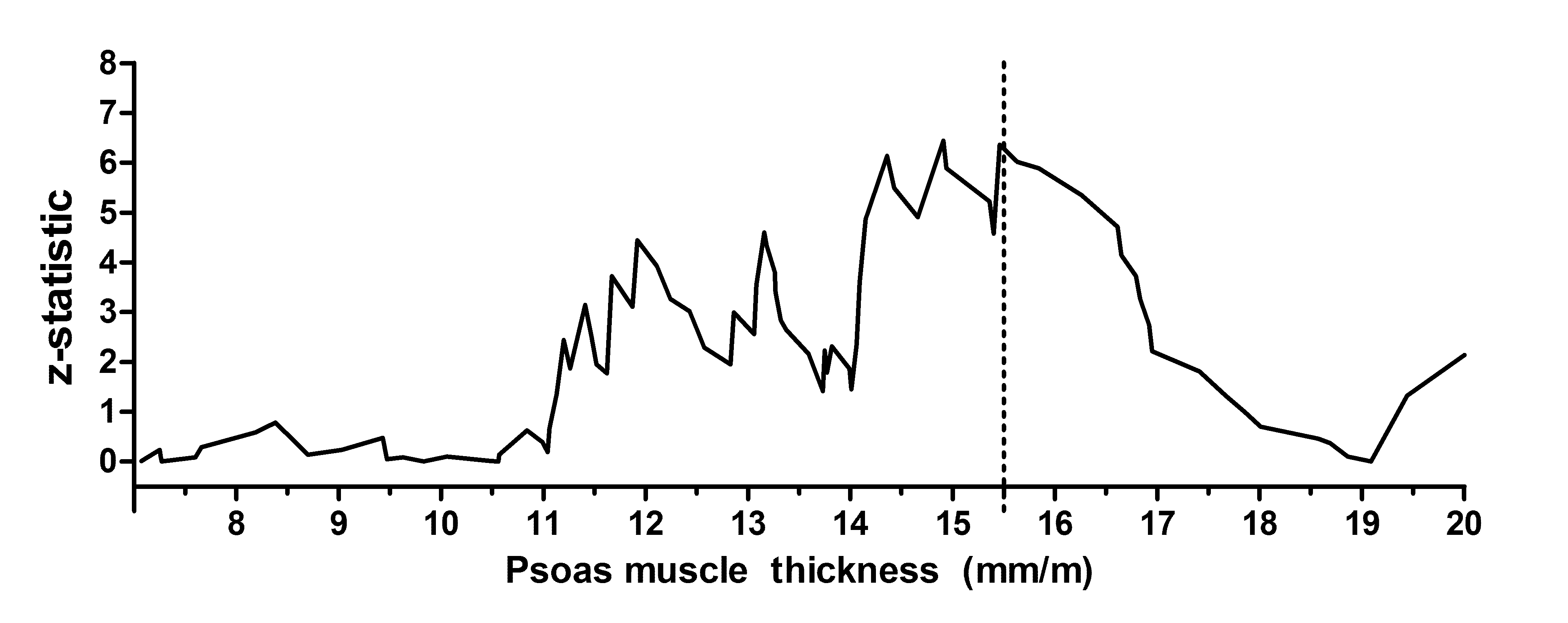


**Supplementary Fig. S1.** The degree of statistical difference in post-transplant hepatocellular carcinoma recurrence between high risk and low risk groups, as stratified by various cutoff values for preoperative psoas muscle thickness (X-axis) was tested by Gray's test statistic (Y-axis).

**Supplementary Table S1.** Comparison of baseline characteristics between patients with an available computed tomography image and patients without an available computed tomography image.

|  | With available CT  (n = 92) | No available CT  (n = 43) | p |
| --- | --- | --- | --- |
| Graft factor |  |  |  |
| Donor age (years) | 29 (22-38) | 28 (22-40) | 0.728 |
| Male donor | 62 (67.4) | 33 (76.7) | 0.268 |
| Graft-to-recipient weight ratio (%) | 1.01 (0.87-1.21) | 0.99 (0.88-1.14) | 0.001 |
| Macrosteatosis ≥5% | 42 (45.7) | 23 (53.5) | 0.396 |
| Cold ischemia time (minutes) | 85 (66-105) | 78 (59-100) | 0.199 |
| Recipient factors |  |  |  |
| Age (years) | 53 (50-58) | 52 (47-57) | 0.164 |
| Body mass index ≥25 kg/m^2^ | 34 (37.0) | 22 (51.2) | 0.119 |
| Diagnosed diabetes | 33 (35.9) | 16 (37.2) | 0.880 |
| MELD score ≥20 | 10 (10.9) | 6 (14.0) | 0.606 |
| Sodium level (mmol/L) | 140 (136-142) | 140 (138-142) | 0.220 |
| Hepatic encephalopathy |  |  | 0.757 |
| None | 81 (88.0) | 39 (90.7) |  |
| Grade I-II (vs. none) | 11 (12.0) | 4 (9.3) |  |
| Refractory ascites | 15 (16.3) | 6 (14.0) | 0.725 |
| Pretransplant tumor treatment history | 72 (78.3) | 24 (55.8) | 0.007 |
| High sensitive C-reactive protein (mg/L) | 0.38 (0.16-1.11) | 0.23 (0.10-0.83) | 0.211 |
| Neutrophil-to-lymphocyte ratio | 1.97 (1.30-3.04) | 1.67 (1.22-2.53) | 0.269 |
| Transverse psoas muscle thickness (mm/m) | 13.1 (10.9-15.3) | 13.7 (10.9-15.7) | 0.594 |
| Sarcopenia | 72 (78.3) | 29 (74.4) | 0.627 |
| Surgical factors |  |  |  |
| Operative time >10 hours | 40 (43.5) | 12 (27.9) | 0.083 |
| Perioperative RBC transfusion >6 units | 27 (29.3) | 14 (32.6) | 0.705 |
| Tacrolimus trough concentration >10 ng/mL | 42 (45.7) | 19 (45.2) | 0.964 |
| Tumor biology |  |  |  |
| Alpha-fetoprotein >100 ng/mL | 35 (38.0) | 20 (46.5) | 0.351 |
| Tumor number |  |  | 0.271 |
| Solitary | 9 (9.8) | 1 (2.3) |  |
| 2-3 (vs. solitary) | 21 (22.8) | 9 (20.9) |  |
| >3 (vs. solitary) | 62 (68.5) | 33 (76.7) |  |
| Tumor size >3 cm | 52 (56.5) | 20 (46.5) | 0.277 |
| Microvascular invasion | 56 (60.9) | 30 (69.8) | 0.316 |
| Bile duct invasion | 6 (6.5) | 1 (2.3) | 0.430 |
| Edmonson grade III-IV (vs. I-II) | 8 (8.7) | 4 (9.3) | 0.908 |
| Nontumor liver cirrhosis | 79 (85.9) | 42 (97.7) | 0.037 |

Data are presented as median (25th percentile, 75th percentile) or frequency (percent). Sarcopenia was defined as transverse psoas muscle thickness <15.5 mm/m.
